# Supplementary material for: Surface Marker Expression in Small and Medium/Large Mesenchymal Stromal Cell-Derived Extracellular Vesicles in Naive or Apoptotic Condition Using Orthogonal Techniques
Source: Cells. 2021 Oct 29;10(11):2948. doi: 10.3390/cells10112948 (PMC8616318; doi:10.3390/cells10112948)
Supplement: Supplementary file 1 [file cells-10-02948-s001.zip › cells-1430370-supplementary.pdf]

Surface Marker Expression in Small and Medium/Large Mesenchymal Stromal Cell-Derived Extracellular Vesicles in Naive or Apoptotic Condition Using Orthogonal Techniques

Renata Skovronova [1], Cristina Grange [2], Veronica Dimuccio [1], Maria Chiara Deregibus [3], Giovanni Camussi [2] and Benedetta Bussolati [1,*]

[1] Department of Molecular Biotechnology and Health Sciences, University of Turin, 10126 Turin, Italy; renata.skovronova@unito.it (R.S.); veronica.dimuccio@unito.it (V.D.).

[2] Department of Medical Sciences, University of Turin, 10126 Turin, Italy; cristina.grange@unito.it (C.G.); giovanni.camussi@unito.it (G.C.).
[3] 2i3T Business Incubator and Technology Transfer, University of Turin, 10126 Turin, Italy; mariachiara.deregibus@unito.it.

* Correspondence: benedetta.bussolati@unito.it; Tel.: +011-6706453

Supplementary Methods

Apoptosis assay

Cytofluorimetric evaluation of apoptotic cells was performed using the Muse™ Annexin V & Dead Cell Kit (Merck-Millipore, Burlington, MA, USA), according to the manufacturer's instructions. Briefly, 10×10^3 naïve cells or cells were starved with RPMI medium with or without addition of 500ng of anti-Fas targeted antibody for 6, 16 or 24h. Cells were then detached and resuspended in Muse™ Annexin V & Dead Cell Kit (Luminex, Austin, TX, USA), and the percentage of apoptotic cells (Annexin V+) was detected.

Flow cytometry

Flow cytometry analysis of the ApoBDs was performed using BD FACSCelesta™ Flow Cytometer. ApoBDs were compared with apoptotic MSCs cells and latex beads of size 4 µm stained with FITC Annexin V antibody for 30min at room temperature.

Supplementary Figures

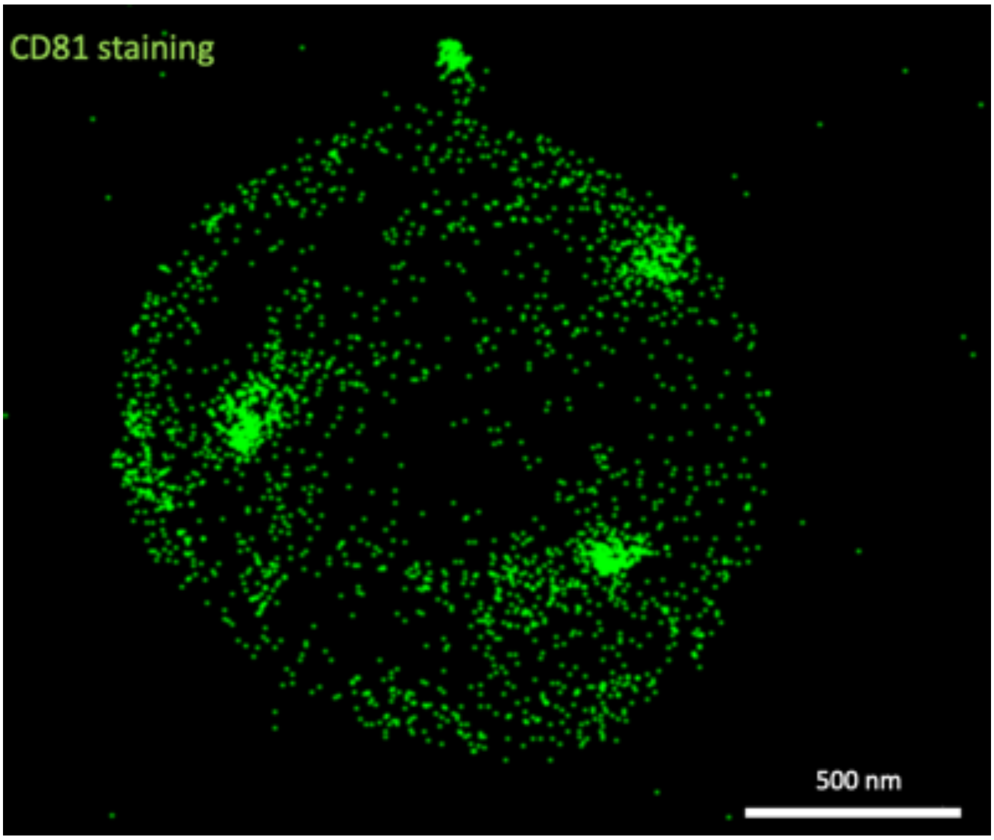

Supplementary Figure S1. Representative super-resolution microscopy images of a single EV from the 10k BM-MSC fraction, showing CD81 tetraspanin surface distribution along the membrane, with areas of condensed expression.

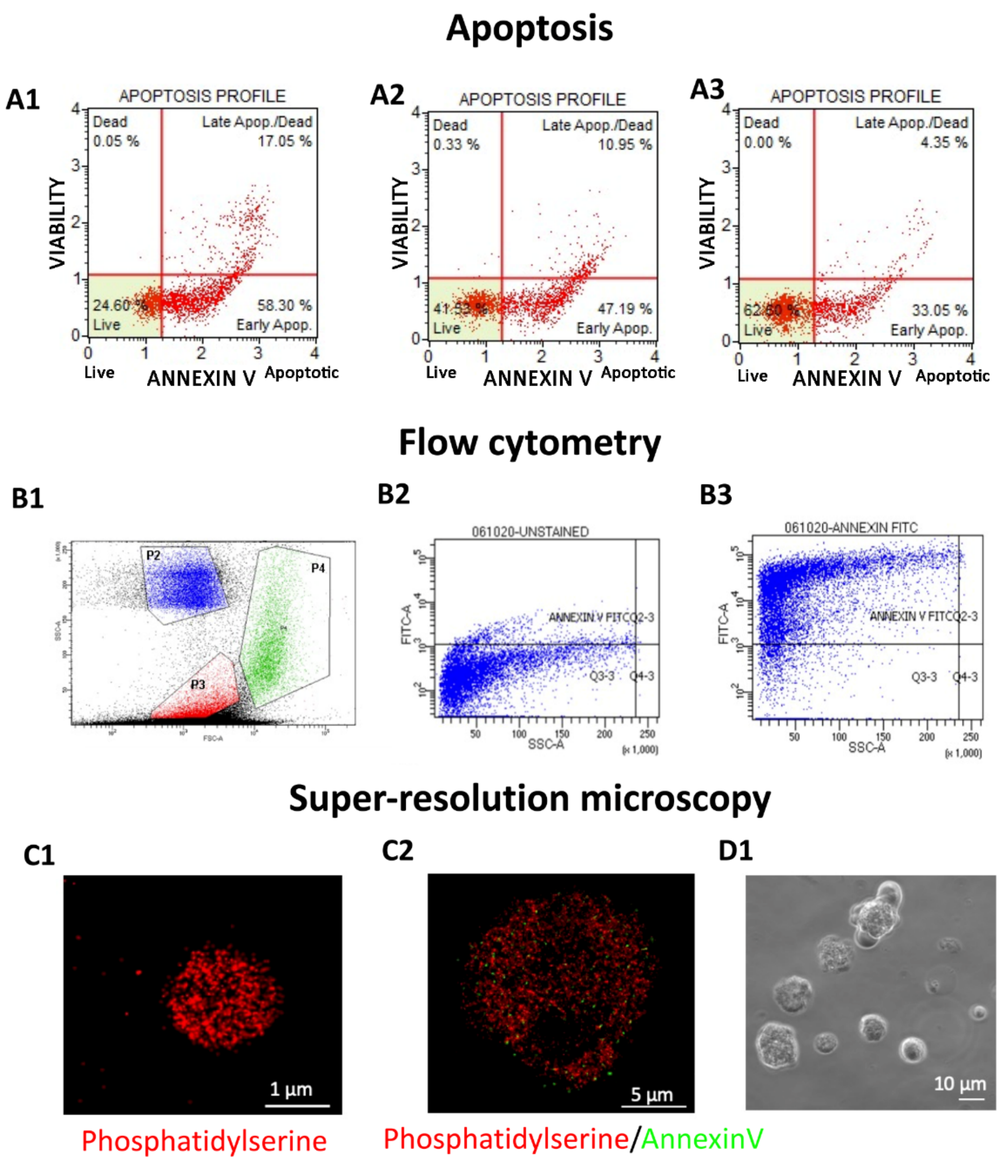

Supplementary Figure S2. Induction of apoptosis and characterization of apoptotic bodies using MUSE flow cytometry and super-resolution microscopy. (A) MSCs viability profile after 24h in RPMI with 500ng of anti-Fas antibody (A1), RPMI (A2) and AlphaMEM with 10%FBS (A3). (B) Representative images of flow cytometric analysis showing Annexin V expression. (B1) flow cytometry gating strategies, P4: apoptotic bodies (ApoBDs), P2: 4 µm latex beads, P3: apoptotic cells. Representative images of flow cytometric analysis showing (B2) unstained ApoBDs and (B3) ApoBDs stained with Annexin V. (C) Representative super-resolution microscopy image of ApoBD fraction stained (C1) with Annexin V (red), (C2) with Annexin V (red) and Phosphatidylserine (green). (D1) Representative bright field microscopy image of ApoBDs realised by MSCs undergoing apoptosis.

Supplementary Table

Supplementary Table S1. MUSE assay used to set up the apoptotic induction. Cells were analysed after 6, 16 and 24h in full condition medium, RPMI or RPMI with 500ng of anti-Fas antibody. Graphical visualization is in supplementary Fig. 2A.

6h	AlphaMEM + 10% FBS	RPMI	RPMI+ anti-Fas	SD
Live	80.45%	71.94%	72.03%	0.07
Early apoptosis	15.95%	23.38%	24.75%	0.07
Late apoptosis	3.50%	4.28%	3.03%	0.01

16h	AlphaMEM 10% FBS	RPMI	RPMI+ anti-Fas	SD
Live	88.69%	81.99%	59.92%	0.01
Early apoptosis	7.07%	13.25%	17.58%	0.02
Late apoptosis	3.89%	4.55%	20.95%	0.00

24h	AlphaMEM 10% FBS	RPMI	RPMI+ anti-Fas	SD
Live	62.60%	41.53%	24.15%	0.01
Early apoptosis	33.05%	47.19%	57.70%	0.01
Late apoptosis	4.35%	10.95%	18.13%	0.02
